# 1-D versus 2-D Entropy Velocity Law for Water Discharge Assessment in a Rough Ditch

**DOI:** 10.3390/e20090638

**Published:** 2018-08-25

**Authors:** Michele Greco, Giovanni Martino

**Affiliations:** 1 Engineering of School, University of Basilicata, 85100 Potenza, Italy; 2 Regional Environmental Observatory Research Foundation of Basilicata, 85052 Marsico Nuovo, Italy

**Keywords:** entropy velocity profile, relative submergence, water discharge

## Abstract

Water discharge assessment in open channel flow is one of the most crucial issues for hydraulic engineers in the fields of water resource management, river dynamics, ecohydraulics, irrigation, and hydraulic structure design, among others. Recent studies state that the entropy velocity law allows expeditive methodology for discharge estimation and rating curve development due to the simple mathematical formulation and implementation. Many works have been developed based on the one-dimensional (1-D) formulation of the entropy velocity profile, supporting measurements in the lab and the field for rating curve assessment, but in recent years, the two-dimensional (2-D) formulation was proposed and applied in studies of regular ditch flow, showing good performance. The present work deals with a comparison between the 1-D and 2-D approaches in order to give a general framework of threats and opportunities related to the robust operational application of such laws. The analysis was carried out on a laboratory ditch with regular roughness, under controlled boundary conditions, and in different stages, generating an exhaustive dashboard for better appraisal of the approaches.

## 1. Introduction

Valuation of water discharge in open channel flow is relevant to hydraulic engineering in several research and operative fields related to irrigation, river monitoring and control, water resource management, hydrological balance assessment and modeling, and calibration of ordinary and advanced runoff and flooding routing models. A truthful discharge evaluation is strongly affected by local hydraulics and geometric characteristics, which can be generally known if flow velocity measurements are available at the same site for different stages. It is better to understand how velocity measurements are expansive, in terms of both time and cost, compared to stage monitoring, which is relatively simple as well. In such a framework, developing operative procedures that can allow simplified commitment in flow measurements is an important strategy for open channel monitoring and control in both natural rivers and artificial ditches. The subject of the present work deals with the definition of a suitable relationship among mean velocity (*U_m_*), maximum velocity (*U_max_*), and velocity ratio (*Φ*(*M*)), to be practically used for water discharge assessment in rectangular ditches operating with limited spot velocity measurements. In such a way, field operators might carry out focused measurements following a robust operative framework, optimizing surveying resources in terms of both time and effort

The general approach follows the results found in recent studies, which outline the possibility of correlating the local flow energy budget to the informational content within the point velocity measurements, adopting a velocity distribution profile derived by an entropy–probabilistic theory. Chiu [1,2] showed a high correlation in one dimension (1-D) between mean and maximum flow velocities through a parameter *M*. Due to the significant implications of such a finding in the field of river monitoring, many authors investigated the trustworthiness of this relationship using field data [3,4,5,6,7]. Overall, they found *M* to be constant at a river site and almost independent from the magnitude of flooding. Therefore, *M* might represent a core parameter of the gauged site, addressing Moramarco and Singh [7], to explore possible relationships between *M* and the hydraulic and geometric characteristics of a river site.

This analysis allows, on the one hand, an explanation of the steadiness of *M*, which does not depend on flood dynamics, such as those expressed by the energy or water surface slope, *Sf*. On the other hand, it allows a definition of the relationship expressing *M* as dependent on the hydraulic radius, Manning’s roughness, and the location of the zero horizontal velocity, generally defined as *y*_0_. For the latter, it was also found that if *y*_0_ is assessed by distinguishing low flows from high flows, then a better estimation of *M* might be obtained across a gauged river site. However, considering that the *y*_0_ location is not a simple assessment and could have high uncertainty, assessment of *M* should be allowed using easy-to-acquire hydraulic and geometric variables, mainly for ungauged river sites. This could be achieved by looking at the relative submergence *D/d* (where *D* is average water depth and *d* is the characteristic dimension of the roughness elements). In natural rivers, actually, the velocity distribution is generally affected by channel geometry, local vegetation, and roughness, and the velocity can be assumed to monotonically increase from 0 at *y*_0_, usually near the bed, to the maximum value at the water surface or closer. In such a case, the velocity distribution may practically be assumed as one-dimensional. Moreover, besides the influence of the boundary, in the case of rectangular channels that are not considerably wide, the velocity varies even along the transverse direction. In such a case, the two-dimensional (2-D) velocity distribution could be taken into account in order to give a better description of flow field, introducing *G* as the 2-D entropy parameter. Furthermore, besides maximum velocity generally occurring at or below the water surface, the dip phenomenon takes place and the aspect ratio (*B/D*, where *B* is channel width and *D* is water depth) [8] influences the position of maximum velocity.

Though *M* depends on hydraulic and geometric characteristics of the flow site [3,7], as well as the mean-to-maximum velocity ratio, *Φ*(*M*), the analyses are intended to assess such a ratio taking into account the main geometric characteristics of flow like roughness and aspect ratio, here defined as flow width/depth. In fact, in the case of open channel flow, Greco [9] demonstrates different behavior of *Φ*(*M*) with respect to the roughness scale. That is, *Φ*(*M*) depends on relative submergence whenever large or intermediate roughness occurs [10]. Thus, the value of *Φ*(*M*) can be derived through the value of the relative submergence, and the mean velocity can be obtained once the maximum velocity is known, operating a spot measurement in the middle kernel of the flow field where it generally occurs. Finally, the results support and validate a robust and fruitful operative chain to be implemented for expeditive water discharge assessment in rough and smooth irrigation ditches at different stages, allowing assessment of the local rating curve.

## 2. Entropy Velocity Profiles in Open Channel Flow

The concept of entropy as a measure of information or uncertainty of a random variable or its probability distribution was formulated by Shannon [11], introducing the principle of entropy maximization (POME) related to the least-biased probability distribution of the random variable constrained by information given previously [12]. The Shannon entropy, or informational entropy, is defined as:

(1) $$\;H=\;-\int_{-\infty \;}^{+\infty }p\left(x\right)logp\left(x\right)dx\;$$

where *p*(*x*) is the continuous probability density function of random variable *x* and represents a quantitative measure of uncertainty associated with a probability distribution of *x* expressed in terms of entropy.

The principle of maximum entropy can be formulated through the method of Lagrange multiplier, obtaining:

(2) $$\;L=-\;\frac{1}{m-1\;}\;\int_{-\infty }^{+\infty }p\left(x\right)\left\{1-\;{\left[p\left(x\right)\right]}^{m-1}\right\}dx+\;\sum_{i=1}^{N}\lambda_{i}\;g_{i}\left(x\right)$$

where *m* is a positive real number, *g*_*i*_(*x*) is the *i*-th constraint function, and *λ_i_* is the Lagrange multiplier for each constraint. The solution of such problem can be carried on in 1-D or 2-D domains, allowing us to obtain two possible flow velocity distributions.

### 2.1. One-Dimensional Velocity Distribution

Chiu [1,2] applied entropy theory to open-channel flows to describe velocity distribution, shear stress, and sediment concentration. The proposed approach analyzes the velocity distribution in the probability domain, supporting the assessment of mean flow velocity and the momentum and energy coefficients, disregarding the shape of cross-sections [2,13].

Let us consider *u* to be time-averaged velocity on the *ξ* iso-velocity curve, monotonically increasing from 0 at *ξ*_0_, corresponding to the channel boundary up to *U_max_* at *ξ_max_*, which may occur at or below the water surface; then, at any value of the spatial coordinate less than *ξ*, the velocity is less than *u*, which can be written in the cumulative distribution function as:

(3) $$\;F(u)\;=\frac{\xi -\xi_{0}}{\xi_{max}-\xi_{0}}$$

Thus, Equation (1) can be written as:

(4) $$\;H=\;-\int_{0}^{U_{max\;}}p\left(u\right)logp\left(u\right)du\;$$

and the probability density function of the velocity distribution is obtained by maximizing the Shannon entropy equation as follows:

(5) $$\;L=\int_{0}^{U_{max\;}}\frac{f\left(u\right)}{m-1}\left\{1-{\left[f\left(u\right)\right]}^{m-1}\right\}du+\;\lambda_{0}\left[\int_{0}^{U_{max}}f\left(u\right)du-1\right]+\lambda_{1}\left[\int_{0}^{U_{max}}uf\left(u\right)du-\bar{u}\right]$$

where *λ*_0_ and *λ*_1_ are the Lagrange multipliers under the following constraint equations:

(6) $$\;C_{1}=\int_{0}^{U_{max\;}}f\left(u\right)du=1\;$$

(7) $$\;C_{2}=\int_{0}^{U_{max\;}}uf\left(u\right)du=\bar{u}$$

(8) $$\;f(u)\;=exp\left(\lambda_{0}-1+\lambda_{1}u\right)$$

Thus, Chiu’s 1-D velocity distribution results as:

(9) $$\;u=\frac{U_{max\;}}{M}ln\left[1+\left(e^{M}-1\right)F\left(u\right)\right]=\frac{U_{max}}{M}ln\left[1+\left(e^{M}-1\right)\frac{\xi -\xi_{0}}{\xi_{max}-\xi_{0}}\right]$$

where *M* is the dimensionless entropy parameter [14,15] and might be used as a uniformity measure of probability and velocity distributions. Thus the value of *M* arises from the mean and maximum velocity values through the following equation:

(10) $$\;\Phi (M)\;=\frac{U_{m}}{U_{max}}=\left(\frac{e^{M}}{e^{M}-1}-\frac{1}{M}\right)$$

which represents the fundamental relationship of the entropy velocity theory, and the assessment of the entropy parameter *M* passes through the knowledge of the ratio between mean and maximum velocities, *Φ*(*M*). The mean velocity value, the location of the mean velocity, and the energy coefficient can be obtained from *M* [14,16]. Further, Equation (10) changes the point of view in terms of the operative employment of entropy theory in the field of open channel flow monitoring and control. A linear relationship between mean and maximum velocities was discovered by collecting the velocity data in some cross-sections of the Mississippi River [17], further remarked upon in several recent studies [3,4,5,6,7,9,13,14,16] and others. In fact, the mean velocity, and thus the discharge, can be obtained once both *Φ*(*M*) and maximum velocity are known, which can be evaluated following several operatively robust approaches.

That is, the relation of *M* from the hydraulic and geometric characteristics of channels in terms of relative submergence and aspect ratio may be obtained following the approach proposed by Greco [9] for *U_m_* as:

(11) $$\;\frac{U_{m}\;}{u_{*}}=\frac{1}{k}ln\frac{D}{d}+\;\frac{1}{k}lnC_{0}$$

where $u_{*}\;$is shear velocity, *d* is characteristic bottom roughness height (*d*_50_ or *d*_84_), *k* is the Von Karman constant, and *C*_0_ is the dimensionless coefficient. Further, the location of maximum velocity from the river bottom, *y_max_*, is of interest, because the maximum velocity does not always occur at the water surface, but at some distance below it, generating the “velocity dip” phenomenon generally related to several factors, one of which is the transverse channel cross-section circulation or secondary currents [18].

In this context, Moramarco and Singh [7] identified the ratio between *U_max_* and u_*_ as:

(12) $$\;\frac{U_{max\;}}{u_{*}}=\frac{1}{k}ln\left(\frac{D}{y_{0}\left(1+\alpha \right)}\right)+\;\frac{\alpha }{k}ln\left(\frac{\alpha }{1+\alpha }\right)$$

with α = (*D/y_max_* − 1). Moreover, Rouse [19] suggested to assume *y*_0_ proportional to the characteristic bottom roughness height d, considering the experimental parameter *C_ξ_ = y*_0_*/d*. Thus Equation (12) becomes:

(13) $$\;\frac{U_{max\;}}{u_{*}}=\frac{1}{k}ln\left(\frac{D}{d}\right)+\;\frac{1}{k}\ln\left(\frac{\alpha^{\alpha }}{C_{\xi }{\left(1+\alpha \right)}^{1+\alpha }}\right)$$

Unlike in Moramarco and Singh [7], the ratio between Equations (11) and (13) clearly proposes *Φ*(*M*) as a function of the relative submergence *D/d*:

(14) $$\;\Phi (M)\;=\frac{U_{m}}{U_{max}}=\frac{ln\left(\frac{C_{0}D}{d}\right)}{ln\left[\frac{D}{d}\;\frac{\alpha^{\alpha }}{C_{\xi }{\left(1+\alpha \right)}^{1+\alpha }}\right]}≅A_{\Phi }ln\frac{D}{d}+B_{\Phi }$$

where $A_{\Phi }$ and $B_{\Phi }\;$are numerical coefficients if Equation (14) has been derived under the assumption that the variability of ln(D/d) ranges from 1 to 10 and the corresponding ratio between the two coefficients, ln$(C_{0})/ln\left(\frac{\alpha^{\alpha }}{C_{\xi }{\left(1+\alpha \right)}^{1+\alpha }}\right)$, is less than 2, as generally occurs in field data. That is, under such limits, the pairs $\left[ln\left(\frac{C_{0}D}{d}\right)/ln\left(\frac{D}{d}\;\frac{\alpha^{\alpha }}{C_{\xi }{\left(1+\alpha \right)}^{1+\alpha }}\right);ln\left(\frac{D}{d}\right)\right]$ can be linearly interpolated (*R*^2^ ≈ 0.9) [13].

Equation (14) outlines the possible influence of bed roughness on the entropy velocity distribution in open channel flows, which depends on the roughness scale according to [10]. Such a dependence between the velocity ratio and relative submergence has been discussed by Greco [9], referring to a large set of data collected both in the field on several rivers and in the laboratory [20,21,22], proposing *Φ*(*M*) ranging in the 0.5–0.9 interval.

### 2.2. Two-Dimensional Velocity Distribution

The analytical two-dimensional approach for the entropy velocity profile [15,23,24] proposes the implementation of Equation (8) in the two-dimensional domain (*x*,*y*), with *y* being the vertical direction starting from the bed upward (positive) and *x* the transverse direction. Thus, *u* = *u*(*x*,*y*) is *f*[*u*(*x*,*y*)], while the cumulative probability distribution function is *f*[*u*(*x*,*y*)].

The partial derivatives of *F*(*u*) with respect to *x_i_* circular coordinates are:

(15) $$\;\frac{\partial F(u)\;}{\partial x_{i}}=\frac{dF\left(u\right)}{du}\frac{\partial u}{\partial x_{i}}=f\left(u\right)\frac{\partial u}{\partial x_{i}}\;\;\;\;$$

From Equation (8), Equation (15) is written as:

(16) $$\;exp\left(\lambda_{1}u\;\right)\frac{\partial u}{\partial x_{i}}=exp\left(1-\lambda_{0}\right)\frac{\partial F\left(u\right)}{\partial x_{i}}\;\;$$

Assuming the quantity w equal to $exp\left(\lambda_{1}u\right)$, Equation (16) can be written as:

(17) $$\;\frac{\partial w\;}{\partial x_{i}}=\lambda_{1}exp\left(1-\lambda_{0}\right)\frac{\partial F\left(u\right)}{\partial x_{i}}\;\;$$

and by the Leibniz rule:

(18) $$\;\int_{\left(0,0\right)}^{\left(x,y\right)}(\frac{\partial w}{\partial x}dx+\frac{\partial w}{\partial y}dy)=w\left(x,y\right)-w\left(0,0\right)$$

The point (0,0) is part of the solution contour domain on which *u*(0,0) = 0, thus Equation (14) becomes:

(19) $$\;\int_{\left(0,0\right)}^{\left(x,y\right)}(\frac{\partial w}{\partial x}\mathrm{dx}+\frac{\partial w}{\partial y}\mathrm{dy})=w\left(x,y\right)-1$$

Considering a generic point of the domain (*x*_0_,*y*_0_) identified by means of a polygonal curve starting from (0,0), ending at (*x*_0_,*y*_0_), and passing through (*x*_0_,0), Equation (19) can be written as:

(20) $$\;\int_{\left(0,0\right)}^{\left(x_{0},y_{0}\right)}(\frac{\partial F\left(u\right)}{\partial y}\lambda_{1}exp\left(1-\lambda_{0}\right)dy+\frac{\partial F\left(u\right)}{\partial x}\lambda_{1}exp\left(1-\lambda_{0}\right)dx)=\lambda_{1}exp\left(1-\lambda_{0}\right)F\left(u\right)$$

Thus, Equation (20) combined with Equation (19) gives:

(21) $$\;\;w\left(x,y\right)=1+\;\;\lambda_{1}exp\left(1-\lambda_{0}\right)F\left(u\right)$$

and with *w*(*x*,*y*) = exp(*λ*_1_*u*), Equation (21) becomes:

(22) $$exp\left[\lambda_{1}u\left(x,y\right)\right]=1+\lambda_{1}exp\left(1-\lambda_{0}\right)F\left(u\left(x,y\right)\right)$$

from which is derived:

(23) $$\;u\left(x,y\right)=\frac{1}{\lambda_{1}}ln\left[1+\;\;\lambda_{1}exp\left(1-\lambda_{0}\right)F\left(u\left(x,y\right)\right)\right]$$

Equation (23) presents the two Lagrange multipliers, *λ*_0_ and *λ*_1_, arising from Equations (6) and (7). Integration of Equation (6) yields:

(24) $$\;\int_{0}^{u_{max\;}}exp\left(\lambda_{0}-1+\lambda_{1}u\right)du=1\;\;\;\to \;\;\;\lambda_{1}exp\left(1-\lambda_{0}\right)=exp\left(\lambda_{0\;}u_{max}\right)-1$$

and posing *λ*_1_*u_max_ = G* as the 2-D entropic parameter [2], Equation (23) can be written as:

(25) $$\;u\left(x,y\;\right)=\frac{u_{max}\;}{G}ln\left[1+\left(e^{G}-1\right)\cdot F\left(u\left(x,y\right)\right)\right]$$

where parameter *G* is determined using the constraint expressed by Equation (7).

Using Equation (25) and the PDF *F*(*u*) defined by Equation (8), the velocity ratio becomes an exponential function of *G* only. Generally, the equation used for deriving velocity distributions, for example Equation (7), refers to the average value of velocity, thus the mean cross-velocity can be computed as:

(26) $$\;\bar{u}=\frac{1}{\Omega }\int_{\Omega }^{\;}\frac{u_{max\;}\;}{G}ln\left[1+\left(e^{G}-1\right)\cdot F\left(u\left(x,y\right)\right)\right]d\Omega$$

That is, if $\bar{u}$ and *u_max_* are known, *G* can be determined by Equation (26), and then the velocity distribution can be calculated using Equation (25), once the Cumulative Density Function (CDF) in 2-D is defined.

In practice, the method requires $\bar{u}$ and $u_{max}$ to be measured.

**Figure 1:**
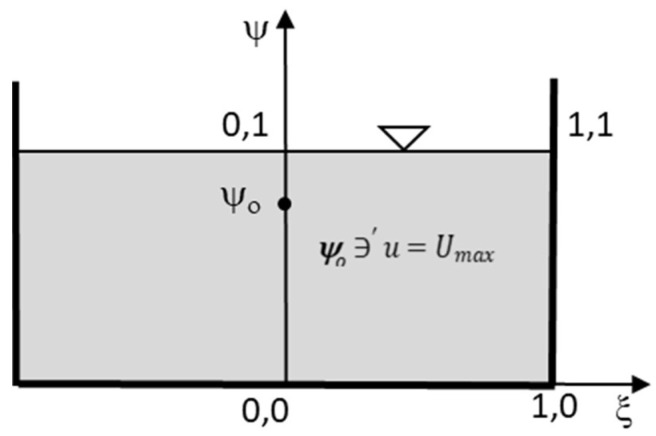
Dimensionless physical domain of rectangular cross-section.

As suggested by Fontana et al. [23], the CDF depends on the geometry of the domain, which is continuous and differentiable within the interval 0–1, and the CDF on the borders is 0 except in one point, where it reaches 1. Thus, consider a rectangular flume in which the velocity distribution is symmetrically distributed with respect to the *y*-axis. One can distinguish the position of coordinates, the location of *U_max_* occurring on or below the water surface depending on distance *y*_0_ of the point at the maximum velocity from the channel bed, and the size of domain *H* (height) and *B*/2 (half width) (Figure 1).

Converting this domain into a dimensionless plane using width and height as normalizing scales and defining the quantities *ψ = y/H*, *ξ = 2x/B*, *ψ*_0_ = *y*_0_*/H*, and *u/U_max_* (Figure 1), Marini et al. [24] suggested to assume *F*(*u*) as:

(27)  F(u)=[1−ξ2]HB·4[(ψ2)ln2ln2−ln|ψ0|− (ψ22 ln2ln2−ln|ψ0|)]

*F*(*u*), given by Equation (27), has two main components depending on *ξ* and the rest depending on *ψ*. That is, as the ratio *H/B* → 0, the first part of *F*(*u*) tends to 1 and so *F*(*u*) depends only on *ψ*. Therefore, as the domain becomes very wide, *F*(*u*) might be assumed depending on just *ψ*, also following a physical intuition. Then, Equation (27) becomes:

(28)  F(u) =4[(ψ2)ln2ln2−ln|ψ0|− (ψ22 ln2ln2−ln|ψ0|)]

Equation (28) gives the 1-D velocity distribution if the maximum velocity is on or below the water surface, and agrees with the result proposed by Chiu [1]. In other words, the 2-D theory formulated by Fontana et al. [23], as represented by Marini et al. [24], when applied to the 1-D case well represents Chiu’s formulation.

Finally, to apply the 2-D velocity distribution equation given by Equation (25), parameter G must be evaluated by means of Equation (27), which represents the value of CFD for mean velocity:

(29) u¯ =umax G ln{1+(eG−1)·[1−ξ2]HB·4[(ψ2)ln2ln2−ln|ψ0|− (ψ22 ln2ln2−ln|ψ0|)]}

## 3. Laboratory Measurements in a Rectangular Ditch

**Figure 2:**
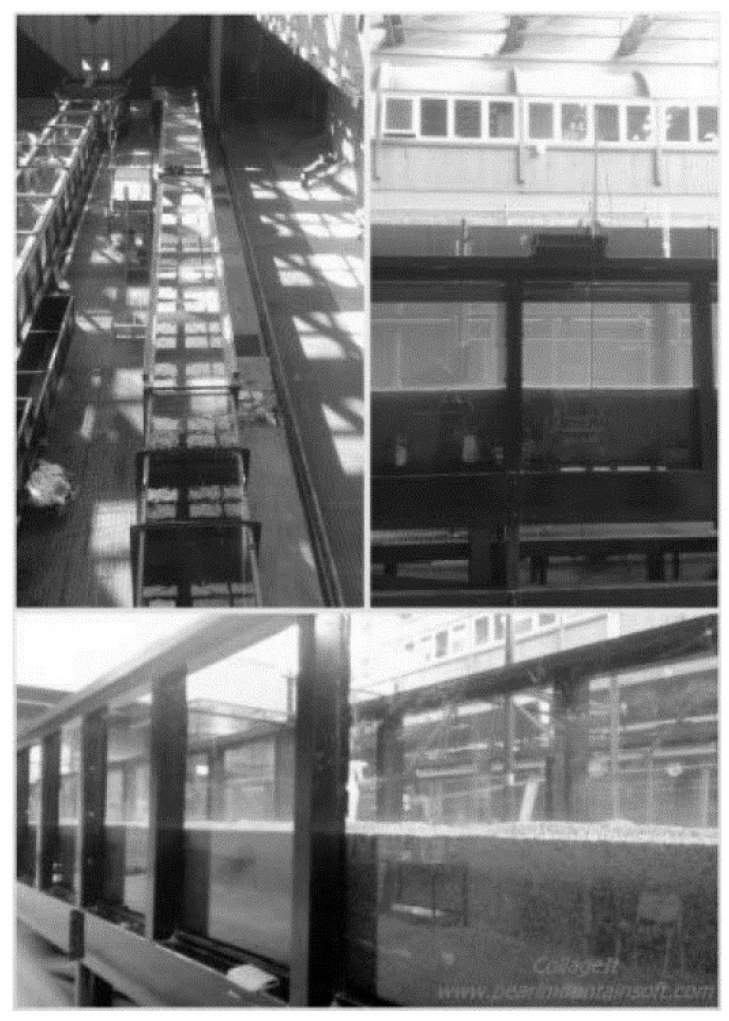
Experimental apparatus for laboratory measurements.

The experimental tests were carried out in the Hydraulics Laboratory of Basilicata University, on two free-surface rectangular flumes. The total length was 9 m for both channels, while the cross-sections were 0.5 × 0.5 m and 1 × 1 m. In both cases, the slope varied from 0% to 1% (Figure 2).

The roughness at the bottom (*d*) was modulated between smooth (0.0005 m roughness height) and rough, the last obtained with a sand bed (0.002 m, standard deviation $\sqrt{d_{84}/d_{16}}=1.67$) and wood spheres 0.035 m in diameter.

The measurement reaches were placed 4 m from the beginning of the flumes, in order to dampen large-scale disturbances and allow a quasi-uniform water depth (the observed differences in water depths upstream and downstream of the measurement reach were always less than 2%). In the end section of the flume, a grid was installed to regulate the water depth for each assigned discharge, to obtain a small longitudinal variation of the flow depth. The experiments were performed in steady flow conditions for different values of discharge (0.015–0.100 m^3^/s) and slope (0.05–1%). The measurement cross-section was located in the middle of the rough measurement reach in order to observe a fully developed flow, avoiding edge effects. The flow depth was measured by two hydrometers placed at the beginning and end of the measurement reach, and water depth, D, was assumed as the average value. Velocity was acquired through a microcurrent meter with a measuring head diameter of 0.01 m, while water discharge was measured with a concentric orifice plate installed in the feed pipe and on a laboratory weir placed at the end of the flumes, and compared to the value calculated according to the velocity-area method [25], with a maximum error of approximately 1 to 2%. In particular, the adopted velocity-area method requires dividing the cross-section area into several verticals (five verticals in the present work) and a subdivision of each vertical into discrete points, in order to assess the mean velocity of the flow along each vertical. The number of measurement points on each vertical (nine points) was chosen in order to have a good reconstruction of the flow field. In fact, in the adopted approach, the difference in velocity between two consecutive points was less than 20%, compared to the higher measured velocity value, and the points close to the channel bottom and the water surface were fixed according to the size of the microcurrent meter.

In such a way, two roughness configurations were enabled: rough rectangular flume (RRF), with relative submergence raging between 1.89 and 6.43; and smooth rectangular flume (SRF), with relative submergence greater than 50.

Table 1 synthetically reports the ranges of variation of the main parameters observed during the experiments for each investigated configuration: water discharge *Q*, water depth *D*, relative submergence *D/d*, aspect ratio *B/D*, and velocity ratio *Φ*(*M*), respectively.

**Figure 3:**
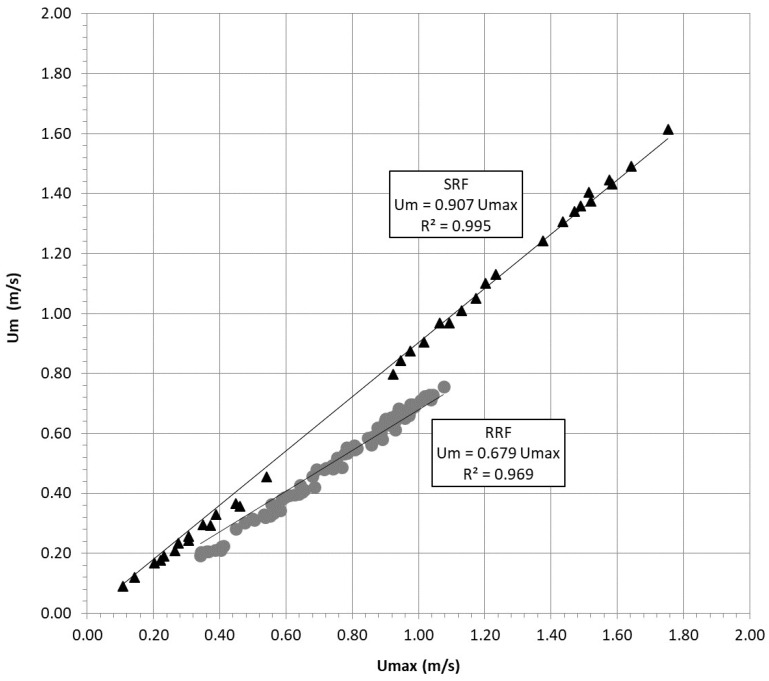
Mean flow velocity vs. maximum velocity observed in laboratory experiments for rough (RRF) and smooth (SRF) channels.

Considering all the verticals measured for each configuration and for all stages, a huge bulk of velocity data was acquired, providing a suitable reconstruction of the flow field. Thus, mean velocities, *U_m_*, and maximum velocities, *U_max_*, were available for further analysis. Figure 3 shows the linear relationship between *U_max_* and *U_m_* for the 2 investigated configurations.

Even if the correlation is very high, with *R*^2^ greater than 0.95, slightly different behavior between smooth and rough bed conditions is immediately recognized. That is, taking into account the cases of SRF, the slope of the interpolating line, which represents the mean-to-maximum ratio *Φ*(*M*), assumes values close to 0.9, while for RRF, the value of *Φ*(*M*) decreases up to 0.67. That is, the dependence of the velocity ratio on roughness, here represented by the relative submergence *D/d* and discussed in the section for Equation (14), seems to be evident and sufficiently confirmed.

**Figure 4:**
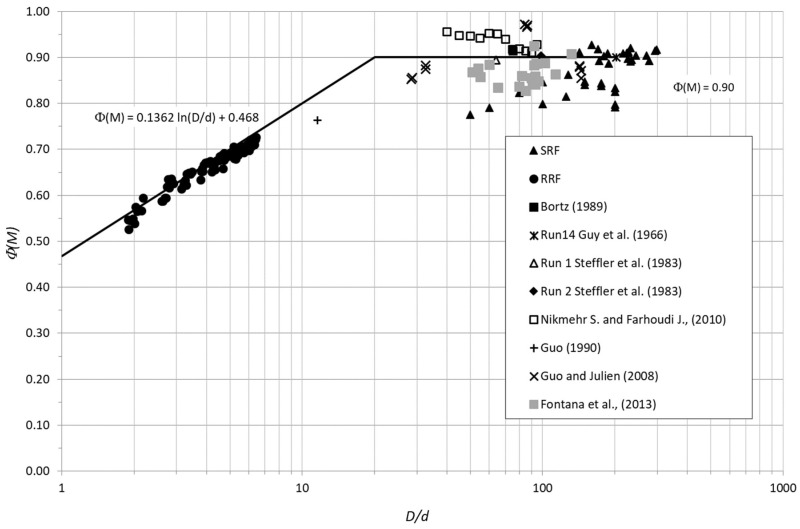
Velocity ratio vs. relative submergence (experiments and literature data).

Figure 4 clearly outlines such an outcome, showing how the velocity ratio is austerely dependent on relative submergence in case of rough flows, while it is sufficiently uniform for values of *D/d* > 20. Furthermore, the same picture proposes data collected by other authors during experimental laboratory studies on smooth and rough flumes [22,26,27,28,29], plotted and compared to those arising from the present research. Figure 4 also deals with robust correspondence between datasets related to the low rough/smooth flow conditions for which the hypothesis of constant value of the mean-to-maximum velocities ratio might be assumed consistent, at least from an operative point of view for *D/d* > 20. At the same time, Equation (14) remains applicable for *D/d* < 20, with the coefficients *A_Φ_* and *B_Φ_* equal to 0.136 and 0.468, respectively (*R*^2^ = 0.95).

These results, including all data available from both present experiments and those from the literature, can be promptly used in the operative chain of water discharge evaluation to obtain the rating curve in a smooth or rough ditch. In detail, let us consider a regular cross-section along a straight channel, where assessing the rating curve could be performed using few velocity measurements in a bulk placed in the middle part of flow on the upper area of the cross-section, where *U_max_* generally occurs [21]. For small roughness (*D/d* > 20, i.e., concrete ditch), the ratio *Φ*(*M*) can be assumed to be 0.9, allowing us to derive the mean velocity/water discharge; and for high/medium roughness flow conditions, Equation (14) (with *A_Φ_* = 0.136 and *B_Φ_* = 0.468) gives the value of *Φ*(*M*), the relative submergence *D/d*, and thus mean velocity.

In more detail, in the case of regular cross-section along a straight channel reach and small roughness (*D/d* > 20, i.e., corresponding to concrete irrigation ditch), rating curve calibration is almost immediate, performing few measurements of velocity in a small volume in the middle of the cross-section on the upper part of the flow, where the maximum velocity is usually expected independent from the aspect ratio, as highlighted in Figure 4. In such conditions, the ratio *Φ*(*M*) can be assumed to be 0.9, allowing us to derive the mean velocity and thus the water discharge. There is further benefit with reduction of measurement time and cost. Once velocity measurements in a cross-section following the above procedure are performed, the observed value of *Φ*(*M*) can suggest whether or not changes in bed roughness occurred.

Finally, use of the entropy velocity profile in 1-D gives robust feedback in terms of operative assessment of water discharge, due to easy and immediate evaluation of the M parameter.

## 4. 1-D vs. 2-D Entropy Velocity Profile: Results and Discussion

The wide set of measurements obtained through the laboratory experiments and the literature allow us to perform a robust comparison between the 1-D and 2-D entropy velocity profiles, in order to obtain suitable information for use in the operative chain for water discharge assessment and computational open channel flow.

Referring to the rectangular cross-section, Equation (9), for the 1-D entropy model, and Equation (25), for the 2-D model, were used on the observed measurement set to provide the flow field modeling. More precisely, Equation (9) was computed on each single vertical, *ψ*, in which the cross-section was divided, while Equation (25) was calculated on the regular grid (*ξ,ψ*) starting from the middle of the cross-section (*ξ* = 0).

**Figure 5:**
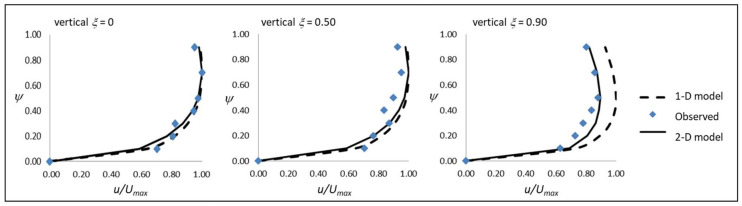
1-D and 2-D entropy velocity profiles applied to the observed velocity dataset.

Furthermore, according to the physical domain adopted in Figure 1, using the dimensionless coordinates, (*ξ,ψ*) and *u/U_max_*, Figure 5 reports the 1-D and 2-D entropy velocity profiles and the observed data as a general example.

As *ξ* increases moving from the middle of the channel toward the border, the 2-D entropy profile always fits the observed data, while the 1-D profile gives a slight overestimation of the local observed velocity, and the difference between the two profiles increases as well. Such behavior of the 2-D entropy velocity profile is physically consistent with the sidewall effect, which induces a no-slip condition on fluid particles while it is implicitly neglected in the 1-D case.

Figure 5 also shows that both velocity profiles present a dip phenomenon; that is, whenever the maximum velocity occurs below the water surface, such a position can be related to the aspect ratio *B/D* whether less than 10 generally.

**Figure 6:**
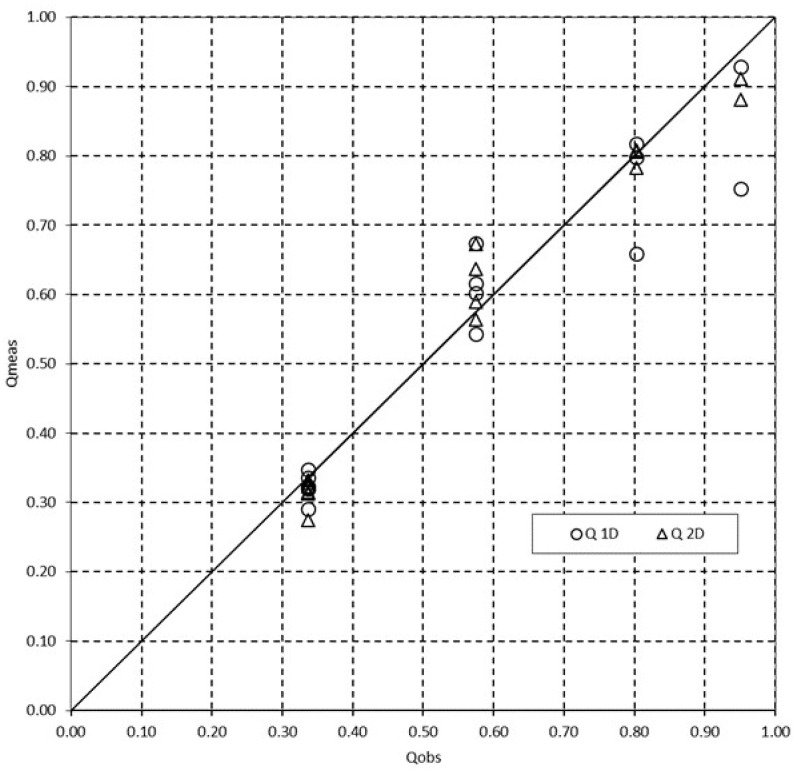
Comparison between measured discharge and discharge obtained with 1-D and 2-D entropy velocity profiles.

Moreover, with respect to water discharge assessment, both models have good performance, as reported in Figure 6, which shows a comparison between the measured discharge and those obtained through 1-D and 2-D entropy velocity profiles.

**Figure 7:**
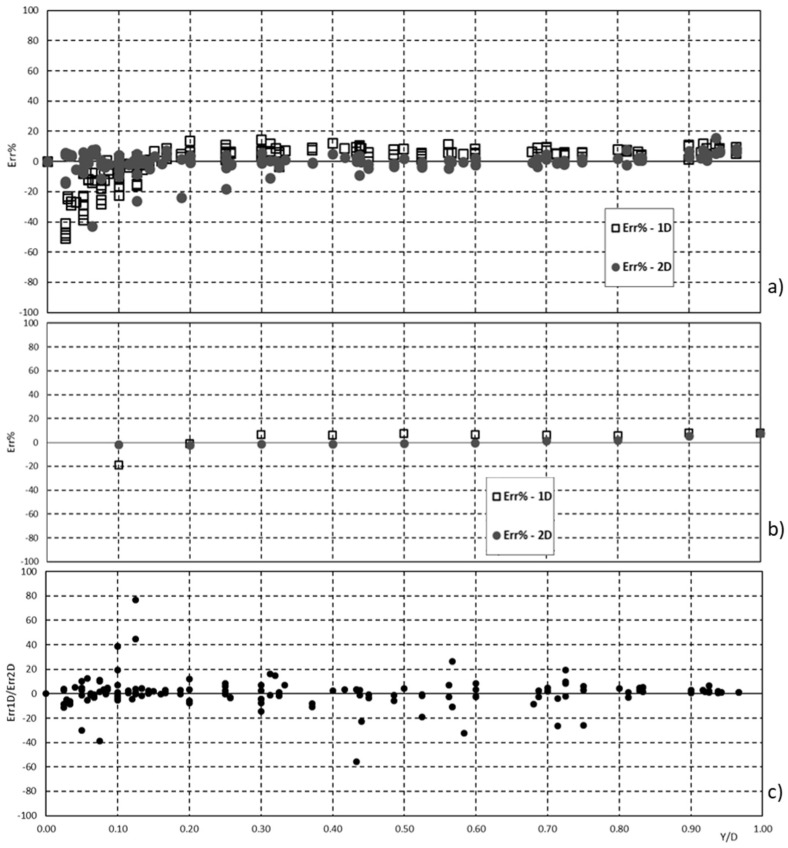
(**a**) 1-D and 2-D percentage error along the vertical; (**b**) cross-section average 1-D and 2-D percentage error along the vertical; (**c**) ratio between 1-D and 2-D percentage error along the vertical.

Figure 7a–c show the 1-D and 2-D percentage error along the vertical, the cross-section average 1-D, and 2-D percentage error along the vertical, and the ratio between 1-D and 2-D percentage error along the vertical, respectively.

From a mathematical point of view, the 2-D approach gives very high performance in terms of error for relatively low values, generally less than 20% all along the vertical, even if the 1-D entropy profile also presents acceptable percentage error for operational activities. On the other hand, the 1-D entropy approach is much easier to derive and apply, providing added value related to the uniform value for high relative submergence, as shown above.

**Figure 8:**
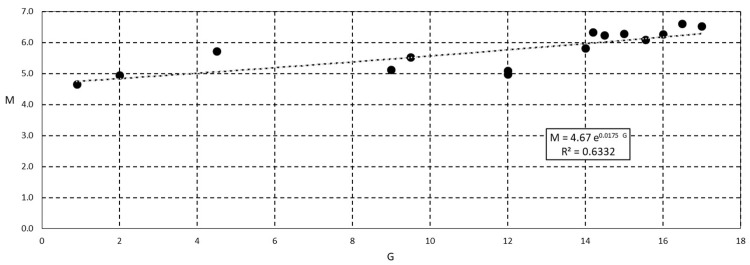
Observed correlation between 1-D entropy parameter *M* and 2-D parameter *G*.

The accurate experimental analysis performed in the laboratory allows us to make a useful contribution regarding a possible correlation between the parameters *M* and *G* in the case of smooth channel. In fact, referring to the set of data collected in the present work for smooth rectangular flume (SRF), Figure 8 suggests the possibility to draw a fruitful correlation between *M* and *G* parameters, proposing an exponential relationship for the observed data:

*M* = 4.67 *e*^0.0175*G*^ (30)

In any case, further analyses, both analytical and experimental, are required to better understand such a correlation, at least for practical use.

Passing through the quick methodologies mentioned above, in which *Φ*(*M*) can be promptly evaluated performing few measurements of velocity in the middle of the cross-section, Equation (30) allow us to assess *G* and then the two-dimensional flow field for further physical and numerical analyses. Considering Equation (14) as suitable to calculate *Φ*(*M*) and using such a value to compute the mean velocity and thus the water discharge *Q_1Dcomp_*, the difference in assessment of water discharge between the 1-D and 2-D entropy profiles reduces. That is, due to the hard work and burden of acquiring a detailed velocity dataset for the 2-D entropy model, the 1-D entropy profile represents a good technical compromise in the chain of water discharge assessment in the field of open channel flow.

**Figure 9:**
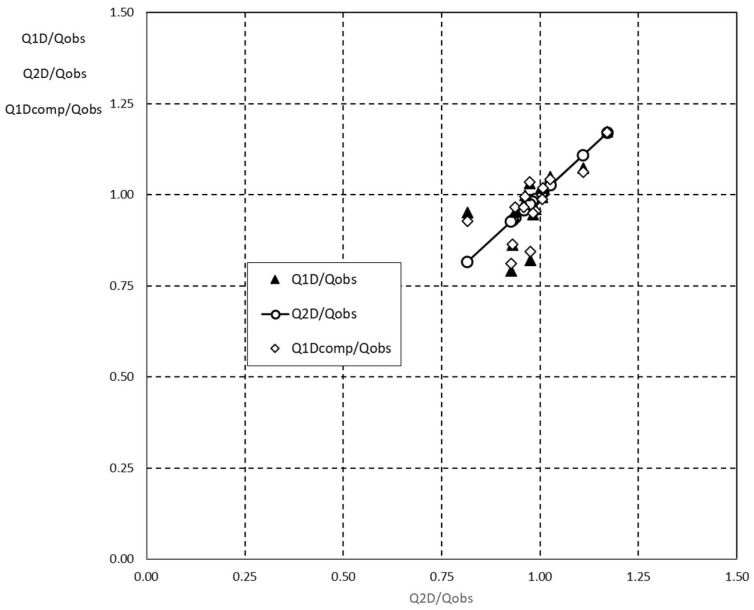
1-D, 2-D, and 1-D computed dimensionless water discharge.

Figure 9 proposes a comparison among the three dimensionless discharges computed as the ratios between them evaluated through the 1-D and 2-D entropy models and the observed discharge, *Q_1D_/Q_obs_*, and *Q_2D_/Q_obs_*, respectively, while for *Q_1Dcomp_/Q_obs_* the values of *M* were obtained through Equation (30). Since the 2-D entropy profile represents the best reconstruction of the flow field, the series of *Q_2D_/Q_obs_* has been assumed as reference to compare the values *Q_1D_/Q_obs_* and *Q_1Dcomp_/Q_obs_*.

The plot implies that the differences among *Q* values are not so relevant and the ratios are strongly close to the unit. In other words, from the operational point of view, use of the 1-D entropy model as a relatively simple way to compute *M* represents a robust and useful methodology or tool for water discharge assessment in ditches, leading to fairly good and suitable results.

## 5. Conclusions

The paper deals with the application of 1-D and 2-D entropy velocity profiles to collected laboratory data on rectangular smooth and rough flumes. The main idea is to better understand the operational difference between the 1-D and 2-D entropy velocity profiles, in order to obtain suitable feedback for water discharge assessment and computational open channel flow in rectangular ditches. That is, due to the possibility of applying expeditive methodology for the assessment of entropy parameter *M*, which can be obtained performing few measurements of velocity in the middle of the cross-section, the 1-D profile, compared to the 2-D profile, represents a good technical and operative compromise in the chain of channel water discharge assessment. Finally, a relationship between *M* and *G* was calibrated among the available experimental data, giving the opportunity to derive a two-dimensional entropy velocity distribution passing though the 1-D approach, which represents a robust and useful methodology for water discharge assessment in channels.

## Figures and Tables

**Table 1:** Ranges of variation for the main parameters of the laboratory experiments.

Type	*Q* (mc/s)	*D* (m)	*D/d*	*B/D*	*Φ*(*M*)
RRF	0.007–0.076	0.07–0.23	1.89–6.43	2.22–7.58	0.52–0.73
SRF	0.025–0.100	0.06–0.40	50–298	2.50-10	0.7–0.93
